# Trip12, a HECT domain E3 ubiquitin ligase, targets Sox6 for proteasomal degradation and affects fiber type-specific gene expression in muscle cells

**DOI:** 10.1186/2044-5040-3-11

**Published:** 2013-05-10

**Authors:** Chung-Il An, Edward Ganio, Nobuko Hagiwara

**Affiliations:** 1 Division of Cardiovascular Medicine, Department of Internal Medicine, University of California, Davis, One Shields Avenue, Davis, CA 95616, USA

**Keywords:** Sox6, Skeletal muscle, Fiber type differentiation, Trip12, E3 ubiquitin ligase, HECT domain, Ubiquitin-proteasome system

## Abstract

**Background:**

A sophisticated level of coordinated gene expression is necessary for skeletal muscle fibers to obtain their unique functional identities. We have previously shown that the transcription factor Sox6 plays an essential role in coordinating muscle fiber type differentiation by acting as a transcriptional suppressor of slow fiber-specific genes. Currently, mechanisms regulating the activity of Sox6 in skeletal muscle and how these mechanisms affect the fiber phenotype remain unknown.

**Methods:**

Yeast two-hybrid screening was used to identify binding partners of Sox6 in muscle. Small interfering RNA (siRNA)-mediated knockdown of one of the Sox6 binding proteins, Trip12, was used to determine its effect on Sox6 activity in C2C12 myotubes using quantitative analysis of fiber type-specific gene expression.

**Results:**

We found that the E3 ligase Trip12, a HECT domain E3 ubiquitin ligase, recognizes and polyubiquitinates Sox6. Inhibiting Trip12 or the 26S proteasome activity resulted in an increase in Sox6 protein levels in C2C12 myotubes. This control of Sox6 activity in muscle cells via Trip12 ubiquitination has significant phenotypic outcomes. Knockdown of Trip12 in C2C12 myotubes led to upregulation of Sox6 protein levels and concurrently to a decrease in slow fiber-specific *Myh7* expression coupled with an increased expression in fast fiber-specific *Myh4*. Therefore, regulation of Sox6 cellular levels by the ubiquitin-proteasome system can induce identity-changing alterations in the expression of fiber type-specific genes in muscle cells.

**Conclusions:**

Based on our data, we propose that in skeletal muscle, E3 ligases have a significant role in regulating fiber type-specific gene expression, expanding their importance in muscle beyond their well-established role in atrophy.

## Background

Mammalian skeletal muscles consist of functionally heterogeneous myofibers, which can be broadly classified into two groups, slow-twitch and fast-twitch fibers. They differ in contraction speed, metabolic capacity, fatigue resistance, sensitivity to calcium, and a variety of other attributes [1-3]. This physiologic diversity among muscle fibers is the outward manifestation of the concerted expression of hundreds of genes unique to each fiber type [4], culminating in the specific biochemical characteristics of either fast fiber or slow fiber type. Despite its significance to muscle physiology and muscle degenerative diseases (e.g., exercise physiology [5]; Duchenne muscular dystrophy [6,7]; inflammatory atrophy [8]), knowledge regarding the molecular mechanisms orchestrating this coordinated expression exists only in vague outlines.

Our recent studies demonstrated that the transcription factor Sox6 directly suppresses transcription of slow fiber-specific genes during mouse muscle development [9,10]. Loss of a functional Sox6 protein in skeletal muscle results in a dramatic increase in slow fibers in fetal as well as in adult mice [9-12]. These observations indicate that Sox6 is one of the key factors regulating the fiber type differentiation of mammalian skeletal muscles.

To uncover how Sox6 activity is controlled in muscle, we searched for regulatory proteins for Sox6 by performing yeast two-hybrid screening. In this manuscript, we report identification of the Trip12 E3 ligase as a negative regulator of Sox6 activity. E3 ubiquitin ligases determine substrate specificity for ubiquitin modification in the ubiquitin-proteasome system [13] and are involved at all levels of transcriptional regulation [14]. We discovered that in C2C12 myotubes, Sox6 protein levels are controlled by the ubiquitin-proteasome system in which Sox6 proteins are polyubiquitinated by Trip12 and degraded by the 26S proteasome. Significant to the orchestrated regulation of muscle fiber phenotype, we found that the suppression of Trip12 levels in C2C12 myotube cultures results in three interlocking events: increased levels of Sox6 protein, downregulation of the slow MyHC isoform (*Myh7*, MyHC-I/β), and upregulation of the fast MyHC isoform (*Myh4*, MyHC-IIb). Our new finding suggests that the ubiquitin-proteasome system plays a significant role in muscle fiber differentiation in addition to its well-established roles in muscle atrophy [15].

## Methods

### Yeast two-hybrid screening

**Figure 1:**
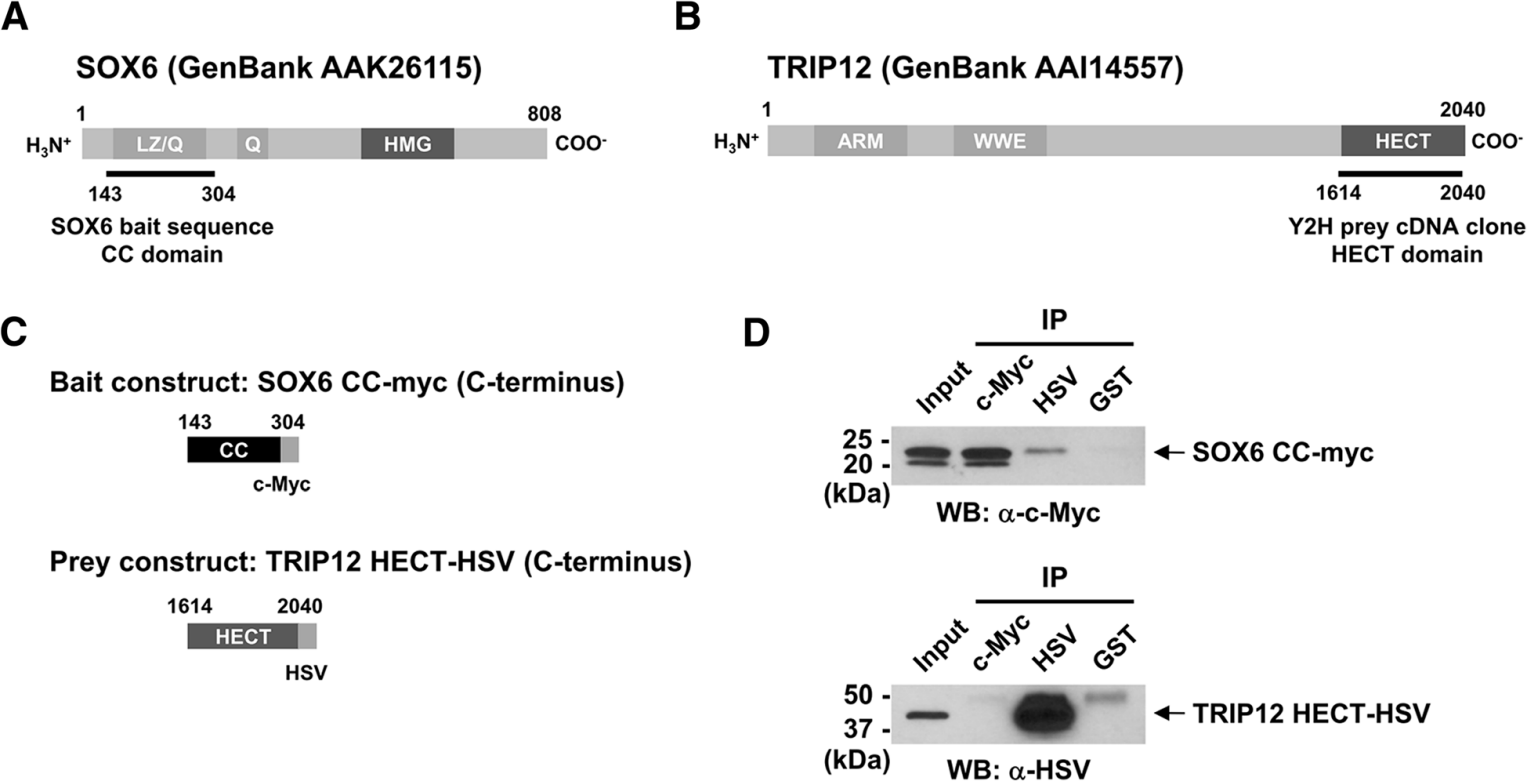
**SOX6 coiled-coil domain physically interacts with TRIP12 HECT domain.** (**A**) Schematic representation of the human SOX6 protein [17]. The coiled-coil (CC) domain containing the leucine-zipper (LZ) motif and Q-box (aa 143–304) was used as bait for screening a human heart cDNA library. The amino acid sequence of the CC domain is 100% conserved between mice and humans. (**B**) Schematic representation of the full-length human TRIP12 protein. The partial TRIP12 cDNA clone identified by yeast hybrid screening contained the most C-terminal HECT domain (aa 1614–2040). (**C**) Diagrams of the bait and prey expression vectors used for Co-IP. The numbers indicate the amino acid regions used for each vector construct. The SOX6 CC-domain was tagged with c-Myc at the C-terminus. The TRIP12 HECT domain was tagged with HSV at the C-terminus. (**D**) Co-IP assays were performed using HEK293 cell lysate transfected with the bait and prey expression vectors depicted in (**C**). Input lanes contained 2% (10 μg) of un-manipulated lysate. Rabbit polyclonal antibodies used for pull down are listed under IP. Mouse monoclonal antibodies used for Western blot (WB) are indicated below each panel. GST antibody was used as a negative control.

The MATCHMAKER Two-Hybrid System 3 (Clontech, Mountain View, CA, USA) was used following the manufacture’s protocol. Construction of SOX6 coiled-coil (CC) domain containing bait plasmid (Figure 1A) was reported previously [16]. AH-109 yeast cells were first transformed with the bait plasmid and subsequently with the human heart cDNA prey plasmid library. Screening was performed on medium-stringency plates (three selection markers, histidine, leucine, and tryptophan) following the manufacturer’s recommendation. Colonies grown to the size of 2–3 mm in 5 days were streaked on high-stringency plates (histidine, adenine, β-gal). Seventy-five large colonies were obtained in this screening, and prey cDNA plasmids were isolated using the YEASTMAKER Yeast Plasmid Isolation Kit (Clontech) for sequencing. Twenty-five of them contained in-frame human protein sequences recorded in GenBank. Of these, the clone containing the C-terminus HECT domain of TRIP12 (Figure 1B) was selected for further analysis.

### Mice

Mice were maintained and killed according to the animal protocol approved by the Institutional Animal Care and Use Committee at the University of California, Davis, which adheres to the National Institutes of Health guidelines.

### Cell culture

HEK293 (human embryonic kidney cell line) and C2C12 (mouse skeletal myoblast cell line) were maintained in growth medium (GM) consisting of Dulbecco’s modified Eagle’s medium (DMEM), 10% fetal bovine serum (FBS), 100 U/ml penicillin, and 100 μg/ml streptomycin at 37°C in a 5% CO_2_-humidified incubator. To induce myotube differentiation, C2C12 cells were incubated in differentiation medium (DM) consisting of DMEM, 2% horse serum, 100 U/ml penicillin, and 100 μg/ml streptomycin.

### Western blotting and densitometric analysis

Brain, heart, lung, kidney, liver, and skeletal muscle (gastrocnemius) were harvested from a 2-month-old wild-type (WT) female mouse, and testis was harvested from a 2-month-old WT male mouse, respectively. Tissue samples were homogenized in RIPA buffer (1X PBS, 1% Nonidet P40, 0.5% sodium deoxycholate, 0.1% SDS) supplemented with protease and phosphatase inhibitor cocktails (Thermo Scientific, Waltham, MA, USA) and clarified by centrifugation at 17,900*g* at 4°C for 15 min. Cultured cell samples were processed as described at each experimental section below. Protein samples were resolved on 7.5% or 4-15% gradient SDS-PAGE gels, transferred to nitrocellulose membranes, and processed for incubation with appropriate antibodies. Signals were detected on X-ray films using Pierce ECL Western Blotting Substrate (Thermo Scientific) or Western Lighting Plus (PerkinElmer, Waltham, MA, USA). For detecting polyubiquitinated Sox6 protein *in vitro* and *in vivo*, WesternBright Sirius Chemiluminescent HRP substrate (Advansta, Menlo Park, CA, USA) was used. The following primary antibodies were used: Sox6 (ab30455, Abcam, Cambridge, MA, USA: rabbit polyclonal), TRIP12 (A301-814A, Bethyl Laboratories, Inc., Montgomery, TX, USA: rabbit polyclonal), β-actin (sc-1616, Santa Cruz Biotechnology, Inc., Santa Cruz, CA, USA: goat polyclonal), TBP (NB500-700, Novus Biologicals, Littleton, CO, USA: mouse monoclonal), HA (H3663, Sigma-Aldrich: mouse monoclonal), c-Myc (R950-25, Invitrogen, Carlsbad, CA, USA: mouse monoclonal), HSV (69171-4, Novagen, Madison, WI, USA: mouse monoclonal), anti-DYKDDDDK (clone L5, 637301, BioLegend, San Diego, CA, USA: rat monoclonal), and myogenin (clone F5D, Developmental Studies Hybridoma Bank, Iowa City, IA, USA: mouse monoclonal).

Quantification of bands was performed using digital scans of the exposed films and ImageJ software (http://imagej.nih.gov/ij/) according to the user guide. Only films with unsaturated intensity of bands were used for densitometric analysis. All densitometry measurements were performed using at least three independent samples, and statistical significance between control and test samples was determined by the two-tailed Student’s t-tests.

### Co-immunoprecipitation (Co-IP)

Interaction between the human SOX6 CC domain and the TRIP12 HECT domain was confirmed by Co-IP. Human SOX6 CC-myc (tagged at the C-terminus) was constructed by inserting the SOX6 CC cDNA sequence into pcDNA3.1/myc-His (Invitrogen, Carlsbad, CA, USA). To generate a C-terminus tagged TRIP12 HECT domain construct (TRIP12 HECT-HSV), the TRIP12 HECT domain cDNA sequence was cloned into pTriEx-1.1 (Novagen, Madison, WI, USA). SOX6 CC-myc and TRIP12 HECT-HSV (Figure 1C) were cotransfected into HEK293 cells using GenJet Plus DNA In Vitro Tranfection Reagent (SignaGen Laboratories, Rockville, MD, USA) and incubated in GM for 48 h. Cells were lysed in Buffer A (50 mM Tris-HCl, pH 7.5, 150 mM NaCl, 1 mM EDTA, 1% Nonidet P40) supplemented with protease and phosphatase inhibitor cocktails (Thermo Scientific) and clarified by centrifugation at 17,900*g* at 4°C for 10 min. IP was performed by incubating 500 μg of protein (in 500 μl Buffer A) with 2 μg rabbit polyclonal antibody targeting c-Myc (A00172, GenScript USA Inc., Piscataway, NJ, USA) or HSV (A00624, GenScript USA Inc.) for 1 h at 4°C on a rocking platform. Anti-GST rabbit polyclonal antibody (ab9085, Abcam) was used as a negative control. The mixture was then supplemented with 20 μl Protein A-agarose beads (Roche, Indianapolis, IN, USA) and incubated overnight at 4°C on a rotating platform. Agarose beads were washed five times with Buffer A, and 25 μl of 2X SDS-PAGE loading buffer was added to the washed agarose beads to extract immunoprecipitated protein. After incubating in boiling water for 5 min, extracted protein was separated on a 4-15% gradient SDS-PAGE gel and analyzed by Western blotting using the antibodies described in the previous section. Co-IP of endogenous Sox6 and Trip12 proteins was performed using nuclear fractions of differentiating C2C12 cells. Nuclear fractions were obtained from C2C12 cells in 15-cm plates differentiated for 48 h in DM using NE-PER Nuclear and Cytoplasmic Extraction Reagents (Thermo Scientific). Protease and phosphatase inhibitor cocktails (Thermo Scientific) were added to appropriate reagents. Prior to IP, the NaCl concentration of nuclear fractions (~400 mM) was adjusted to ~150 mM using Buffer A without NaCl. IP and Western blotting was performed with Rabbit TrueBlot Set (Rockland Immunochemicals, Inc., Gilbertsville, PA, USA) using 300 μg of nuclear protein and 2 μg of rabbit polyclonal antibodies against Sox6 (ab30455, Abcam), TRIP12 (A301-814A, Bethyl Laboratories, Inc.), and GST (ab9085, Abcam) according to the manufacturer’s instructions. Immunoprecipitated protein was separated on a 7.5% SDS-PAGE gel and was analyzed by Western blotting using the same Sox6 and TRIP12 antibodies.

### Tagged protein purification and *in vitro* ubiquitination assay

Full-length human SOX6 cDNA [17] was cloned into pcDNA3.1/myc-his to produce a His-tagged SOX6-myc expression vector, while full-length human TRIP12 cDNA (I.M.A.G.E. clone ID 40083165, ATCC, Manassas, VA, USA) was cloned into pTriEx-1.1 to produce a His-tagged TRIP12-HSV expression vector. HEK293 cells were transfected with each of the tagged protein expression vectors and grown for 48 h. Cells were harvested in Buffer B (50 mM Tris, pH 7.4, 150 mM NaCl, 1 mM EGTA, 1% Nonidet P40, 0.3% sodium deoxycholate) supplemented with 10 mM imidazole, protease, and phosphatase inhibitor cocktails (Thermo Scientific), and His-tagged SOX6 and TRIP12 proteins were purified at 4°C using MagneHis Protein Purification System (Promega Corp., Madison, WI, USA) following the manufacturer’s instructions. After purification, buffer was changed to Buffer A (see the previous section) supplemented with protease and phosphatase inhibitor cocktails (Thermo Scientific) using Amicon Ultra-0.5, Ultracel-30 Membrane, 30 kDa (EMD Millipore, Billerica, MA, USA).

Ubiquitination of SOX6 by TRIP12 *in vitro* was determined using the purified proteins and substrates as described previously [18] with minor modifications. Briefly, 100 ng of ubiquitin-activating enzyme E1 (Enzo Life Sciences, Plymouth Meeting, PA, USA), 250 ng of E2 (UbcH5a, Enzo Life Sciences), 0.8 μg of ubiquitin (Enzo Life Sciences), and 300 ng of purified SOX6 were mixed in 20 μl reaction buffer (25 mM Tris-HCl, pH 7.5, 50 mM NaCl, 5 mM ATP, 10 mM MgCl_2_, 1 mM DTT, 1X protease and phosphatase inhibitor cocktails) with or without 3 μg of purified TRIP12 and were incubated at 37°C for 0, 1, and 2 h. Reactions were terminated by the addition of 20 μl 2X SDS-PAGE loading buffer. Detection of ubiquitinated SOX6-myc protein was performed by Western blot analysis using anti-c-Myc antibody (R950-25, Invitrogen).

### *In vivo* ubiquitination assay

HEK293 cells were plated in 60-mm culture dishes at a density of 2 × 10^6^ cells/plate and incubated for 24 h. Cells were cotransfected with 0.5 μg of HA-Ub vector (obtained from Dr. Aldrin V. Gomes at University of California, Davis), 0.5 μg of Sox6-FLAG vector [mouse full-length Sox6 cDNA was cloned into pcDNA3.1/Zeo(+) (Invitrogen) with a FLAG tag at the C-terminus], and the indicated amount of TRIP12-HSV vector using 7.5 μl of GenJet Plus DNA In Vitro Tranfection Reagent (SignaGen Laboratories). pcDNA3.1/Zeo(+) and pTriEx-1.1 were used as empty vectors for Sox6-FLAG and TRIP12-HSV vectors, respectively. 24 h after incubation, 10 μM MG132 (EMD Millipore) was added to the medium to inhibit the proteasome activity, and cells were incubated for another 6 h. Cells were collected and lysed in Buffer B (see the previous section) containing protease inhibitor cocktail and 10 mM N-ethylmaleimide (an inhibitor for deubiquitinating enzymes), and briefly sonicated using Bioruptor (Diagenode, Denville, NJ, USA). Lysate was cleared by centrifugation at 17,900*g* for 10 min at 4°C, and protein was quantified using BCA Protein Assay Reagent (Thermo Scientific). After pre-clearing 500 μg of total protein using 2 μg of rat IgG2a, κ (BioLegend), and 20 μl of Protein G-agarose beads (Roche), protein samples were mixed with 5 μg of anti-DYKDDDDK (FLAG) tag antibody, and the mixture was incubated for 2 h at 4°C on a rocking platform. Then 20 μl of Protein G-agarose beads was added, and the mixture was incubated overnight at 4°C on a rocking platform. Agarose beads were washed five times with RIPA buffer, and 25 μl of 2X SDS-PAGE loading buffer was added to the washed agarose beads to extract immunoprecipitated protein. After incubating in boiling water for 5 min, extracted protein was separated on a 7.5% SDS-PAGE gel and was analyzed by Western blotting using anti-HA antibody to detect polyubiquitinated Sox6-FLAG protein. The membranes were subsequently incubated with anti-DYKDDDDK (FLAG) tag antibody to detect Sox6-FLAG protein. Input samples were also analyzed using TRIP12 antibody to detect both endogenous TRIP12 and overexpressed TRIP12-HSV proteins.

### siRNA experiments

C2C12 cells were plated in six-well plates at a density of 2 × 10^5^ cells/well and incubated in GM for 24 h. siRNAs (pre-designed DsiRNAs from Integrated DNA Technologies, Coralville, IA, USA) were transfected using 10 μl of TransIT-TKO (Mirus Bio LLC, Madison, WI, USA) at 25 nM final concentration. Then 24 h after transfection, cells were rinsed with PBS once and were incubated in DM for 48 h.

To extract protein, cells were rinsed twice with ice-cold PBS and were treated with trichloroacetic acid (TCA) as described previously [19]. Briefly, cells were incubated in 10% TCA for 30 min on ice and were collected into 1.5-ml tubes. After centrifugation at 17,900*g* for 5 min at 4°C, cell pellets were briefly sonicated in 1X SDS-PAGE loading buffer without bromophenol blue (BPB) and 2-mercaptoethanol (2-ME). After quantification, a hint of BPB and 2-ME (5%) were added, and the protein samples were incubated in boiling water for 5 min. An equal amount of protein was separated on a 7.5% or 4-15% gradient SDS-PAGE gel and analyzed by Western blotting using Sox6, TBP, TRIP12, and β-actin antibodies.

For RNA extraction, cells were lysed in TRIzol reagent, and total RNA was extracted using Direct-zol RNA MiniPrep (Zymo Research, Irvine, CA, USA) with DNase I treatment steps according to the manufacturer’s instructions. After synthesizing cDNA using High Capacity cDNA Reverse Transcription Kit (Applied Biosystems, Carlsbad, CA, USA), quantitative PCR (qPCR) was performed on ABI Prism 7900HT Sequence Detection System (Applied Biosystems) using PrimeTime qPCR Assays (Integrated DNA Technologies) or TaqMan Gene Expression Assay (Applied Biosystems) and SensiFast Probe Hi-ROX Kit (Bioline, Taunton, MA, USA). Results were normalized to the β-actin (*Actb*) transcript level, and all statistical analyses were performed using the two-tailed Student's t-tests. For the time course experiments using differentiating C2C12 cells, *Huwe1* and *Tbp* were used as reference genes instead of *Actb* because reference gene analysis using GeNorm [20] revealed that the combination of these two genes represented the most stably expressed genes in differentiating C2C12 cells among eight genes tested (*Actb*, *Gapdh*, *Huwe1*, *Lrrc40*, *Rpl37a*, Rpl41, *Tbp*, and *Ubb*: data not shown). Additional analysis using NormFinder [21] also showed that these two genes are stably expressed in differentiating C2C12 cells (data not shown). To use these two genes as a reference, raw threshold cycle (C_T_) values of these two genes were averaged according to the instructions of NormFinder, and the mean C_T_ values were used for further calculations. Information on siRNAs, PrimeTime qPCR Assays, and TaqMan Gene Expression Assay used is provided in Additional file 1: Table S1.

### Treatment with cycloheximide and MG132

C2C12 cells were plated in six-well plates at a density of 3 × 10^5^ cells/well and incubated in GM for 24 h. Cells were rinsed with PBS once and incubated in DM for 24 h (for time-course experiments) or 48 h (for MG132 treatment). For time-course experiments, 100 μg/ml cycloheximide (CHX) was added to DM, and cells were incubated for 0, 6, 12, and 24 h. For MG132 treatment, 1 μM MG132 was added to DM together with 100 μg/ml CHX, and cells were incubated for 6 h. As a negative control, the same volume of dimethyl sulfoxide (DMSO) was added. Protein was extracted using TCA as described above and analyzed by Western blotting using appropriate antibodies.

## Results

### SOX6 interacts with TRIP12, an E3 ubiquitin ligase expressed in muscle

During the formation of healthy muscle tissue, Sox6 functions to suppress transcription of a wide variety of slow fiber-specific genes [9,12]. To identify the proteins that interact with Sox6 and regulate the muscle fiber phenotype, we performed yeast two-hybrid screening. For the bait, the coiled-coil domain (CC domain) including the leucine zipper (LZ) motif and glutamine-rich domain (Q-box) of human SOX6 was used (Figure 1A) [17]. This choice of bait was based on the following rationales: (1) the coiled-coil domain is a known protein-protein interacting domain shown to interact with multiple proteins [16,22-26], and (2) the amino acid sequence and location of this domain are 100% conserved between the mouse Sox6 and human SOX6 [17,27]. With this bait, we screened a cDNA library of adult human heart, where SOX6 is moderately expressed [17]. One of the candidates isolated was a clone containing the C-terminus part of TRIP12 (Figure 1B). Currently, what is known about this protein can be summarized succinctly: TRIP12 belongs to the HECT family of E3 ubiquitin ligases, a family characterized by their highly conserved catalytic subunit located at the C-terminus (HECT domain) [28]. Originally identified as a protein that interacts with the ligand-binding domain of the thyroid hormone and retinoid receptors [29], its function as an E3 ubiquitin ligase was reported soon after for multiple targets [18,30-32]. Additionally, it was shown to be essential for mammalian development, as evidenced by a report showing that targeted inactivation results in embryonic lethality in mice [33]. Although Trip12’s function in muscle has not been reported, we hypothesized that the presumed regulation of Sox6 activity by Trip12 would significantly affect muscle differentiation and therefore we decided to investigate.

To confirm the physical interaction between the SOX6 bait and the TRIP12 prey proteins, we first performed Co-IP using lysates from HEK293 cells cotransfected with expression vectors for the bait and prey tagged with c-Myc and HSV epitopes at their C-termini, respectively (Figure 1C). As shown in the upper panel of Figure 1D, the SOX6 bait protein was successfully co-immunoprecipitated with the TRIP12 prey protein when an antibody for the TRIP12 prey (anti-HSV antibody) was used for IP. However, the TRIP12 prey protein was not co-immunoprecipitated when the SOX6 bait protein was pulled down with a c-Myc antibody (Figure 1D, lower panel). The same results were observed when the SOX6 bait protein was fused with a FLAG tag at the N-terminus and anti-FLAG antibody was used for IP (data not shown). These results suggest that both the N- and C-termini of the SOX6 bait protein are physically blocked by the TRIP12 prey protein or unknown protein(s) in HEK293 cells and therefore are not available for Co-IP of the TRIP12 prey protein by the SOX6 bait.

**Figure 2:**
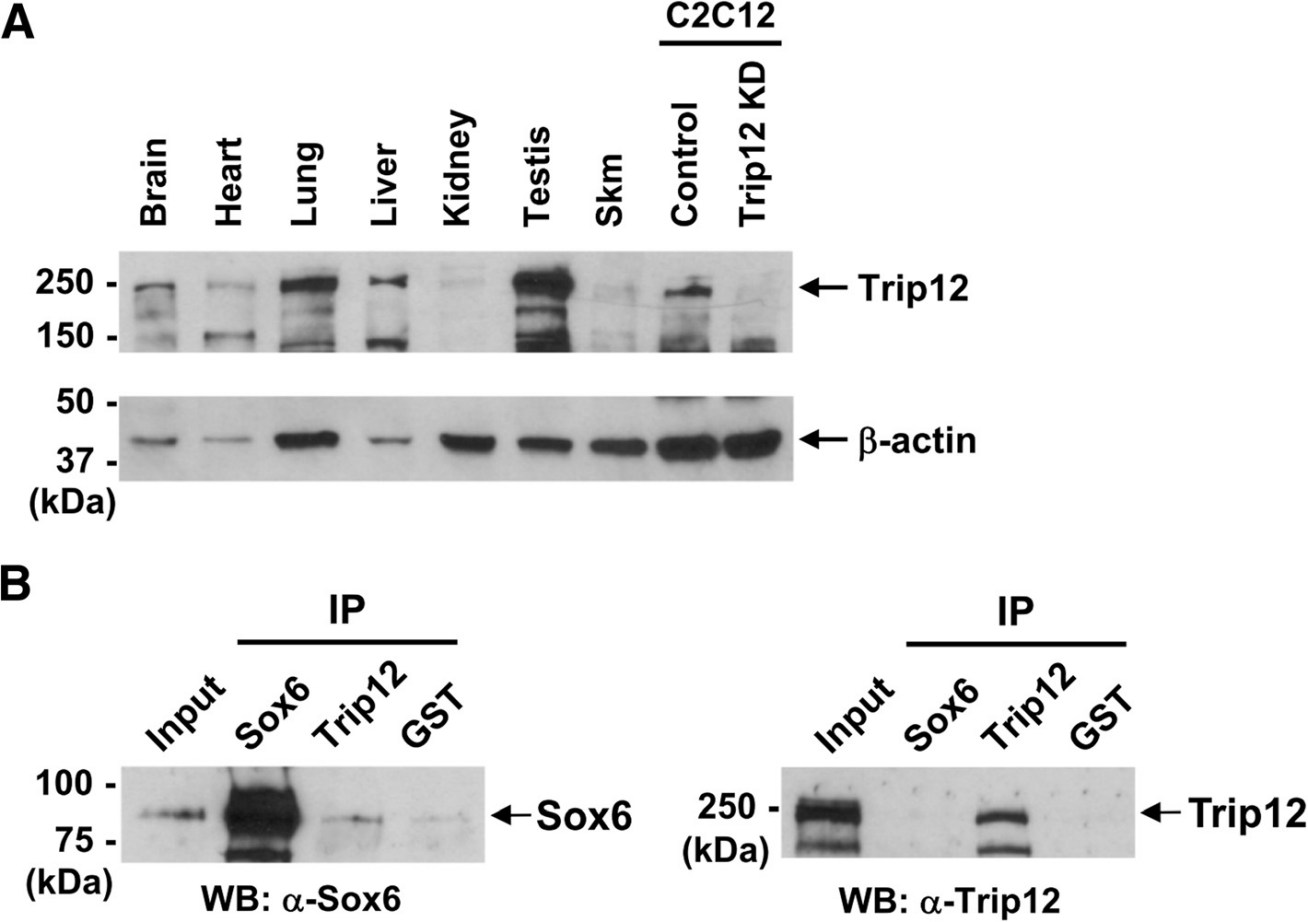
**Trip12 protein expression in adult mouse tissues and interaction between endogenous Sox6 and Trip12 proteins.** (**A**) Western blot analysis was performed to determine Trip12 protein expression in adult mouse tissues. Each lane contained 30 μg of protein except for the C12C12 samples, which contained 15 μg of protein per lane. Relevant protein size markers (kDa) are indicated to the left. β-actin was used as a loading control. (**B**) Co-IP of endogenous Sox6 and Trip12 proteins using nuclear fractions of differentiating C2C12 cells. Input lane contained 5% (15 μg) of pre-cleared nuclear protein. Antibodies used for pull down are listed under IP, and antibodies used for Western blot (WB) are indicated below each panel. GST antibody was used as a negative control.

Next, since little is known about Trip12 expression in the mouse, we investigated expression of the Trip12 protein in adult mouse tissues using Western blotting. The Trip12 band (the full-length mouse protein is approximately 224 kDa) was detected in various tissues including skeletal muscle (Figure 2A). The specificity of the antibody and the actual size of Trip12 band detected were confirmed using Trip12 siRNA, which specifically decreased the signal of the ~250 kDa band (Figure 2A). Since Sox6 protein is expressed in both adult heart and skeletal muscle [17,34] as well as in the nuclear fraction of C2C12 cells (unpublished data), we examined whether the endogenous Sox6 and Trip12 proteins also directly interact. Co-IP using nuclear fractions of differentiating C2C12 cells showed that endogenous Sox6 was co-immunoprecipitated with endogenous Trip12 when Trip12 antibody was used for IP (Figure 2B, left panel), indicating physical interaction of these proteins in C2C12 cells. However, endogenous Trip12 was not co-immunoprecipitated when Sox6 antibody was used for IP (Figure 2B, right panel), suggesting that the C-terminus of endogenous Sox6 protein, where an epitope for the Sox6 antibody used is located, is physically blocked by Trip12 or unknown protein(s). Since the pull-down using both tagged-proteins (Figure 1D) and the endogenous proteins (Figure 2B) gave us similar results, i.e., the antibodies detecting Sox6 proteins did not pull down Trip12 proteins, binding of Trip12 to Sox6 may result in masking a large surface area of the Sox6 protein.

### TRIP12 polyubiquitinates Sox6

In the ubiquitin-proteasome system, E3 ubiquitin ligases such as Trip12 facilitate the transfer of ubiquitin to specific substrates [13]. To examine whether Sox6 is a substrate of TRIP12 E3 ligase activity and if this interaction results in polyubiquitination of Sox6, we performed both *in vitro* and an *in vivo* ubiquitination assay.

**Figure 3:**
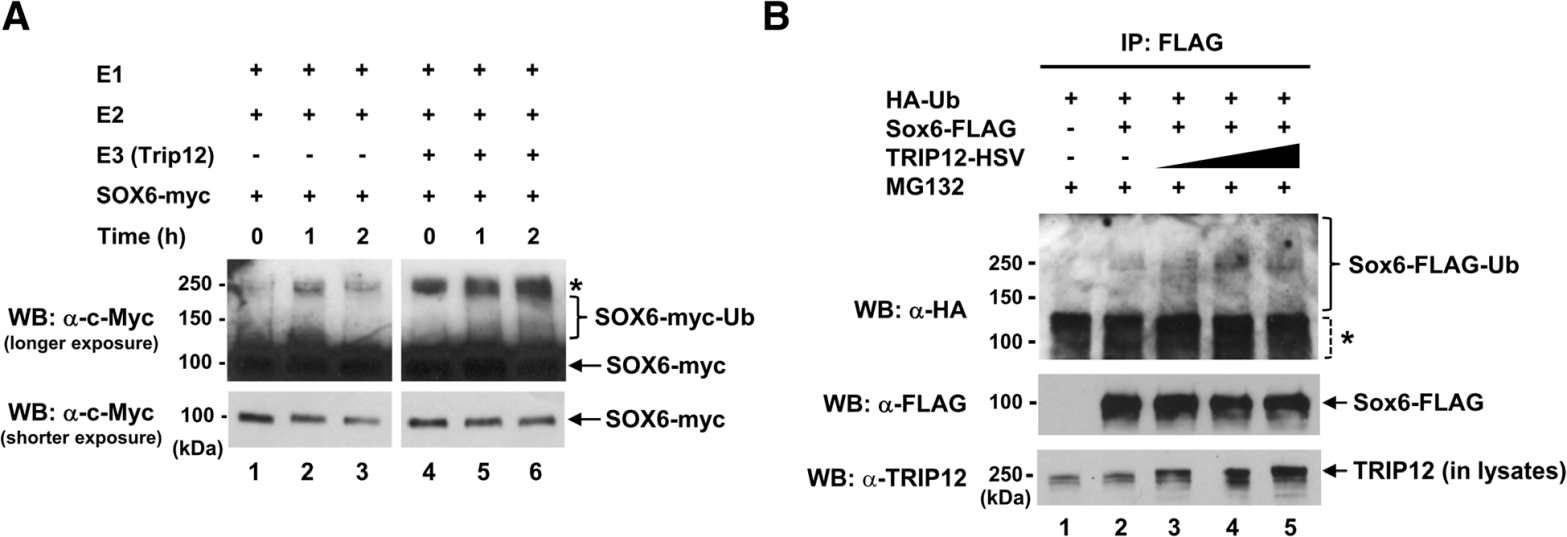
**TRIP12 ubiquitinates Sox6 *in vitro *and *in vivo*.** (**A**) *In vitro* ubiquitination of SOX6 protein was performed using purified SOX6-myc as the substrate and TRIP12 along with the combination of enzymes as indicated in the figure (see the Methods section for details). Lanes 4–6 contain 3 μg of TRIP12-HSV. Western blot (WB) was performed using c-Myc antibody to detect the degree of ubiquitination of Sox6-myc. *Asterisk* indicates non-specific bands detected in all reactions. (**B**) Sox6 is ubiquitinated by TRIP12 *in vivo*. HEK293 cells were cotransfected with plasmid DNAs encoding HA-Ub, Sox6-FLAG, and increasing amounts (0.5, 1, and 1.5 μg) of TRIP12-HSV, and lysates were immunoprecipitated (IP) with anti-DYKDDDDK (FLAG) antibody, and then processed for Western blotting (WB) using anti-HA antibody. *Asterisk* indicates non-specific bands detected in all IP samples, although it is possible that these bands also contain ubiquitinated Sox6-FLAG protein of lower molecular weights (with ~1 to 4 ubiquitin moieties) in Sox6-FLAG-transfected samples. The same membrane was subsequently incubated with anti-DYKDDDDK (FLAG) antibody to detect Sox6-FLAG protein; 10 μg (2%) of input protein samples (lysates) was also subjected to Western blotting using anti-TRIP 12 antibody to detect both endogenous TRIP12 and overexpressed TRIP12-HSV proteins.

We first performed an *in vitro* ubiquitination assay using purified recombinant proteins. The process of ubiquitination of a target protein involves three enzymes, a ubiquitin-activating enzyme, E1, a ubiquitin-conjugating enzyme, E2, and a ubiquitin-protein ligase, E3 [13,28]. In addition, E4 enzymes have recently been shown to enhance the polyubiquitination reaction in some cases [35]. To reconstitute the ubiquitination reaction, an equal amount of purified SOX6-myc (substrate) was combined with the representative E1 and E2 enzymes along with the full-length TRIP12 E3 ligase (see the Methods section). As shown in Figure 3A, addition of TRIP12 resulted in a greater level of ubiquitination of SOX6-myc protein in a time-dependent manner detected as an increasing upward smear (lanes 4, 5, and 6). To test if this process occurred *in vivo*, we performed *in vivo* ubiquitination assays. HEK293 cells were cotransfected with the full-length TRIP12-HSV, Sox6-FLAG, and HA-tagged ubiquitin (HA-Ub) expression vectors. Immunoprecipitation was performed with FLAG antibody to concentrate Sox6-FLAG protein, followed by Western blotting using HA antibody to detect ubiquitinated Sox6-FLAG protein. As TRIP12 protein expression was doubled and tripled, the amounts of high-molecular-weight products, which are the sign of multiple additions of HA-Ub molecules, also increased (Figure 3B), reflecting the addition of multiple ubiquitin molecules to the Sox6-FLAG substrate. As would be expected from a fixed pool of substrate, the non-ubiquitinated Sox6-FLAG protein levels decreased (Figure 3B), indicating that more Sox6-FLAG was polyubiquitinated as Trip12 levels increased.

### Knockdown of Trip12 in C2C12 myotubes results in a concurrent increase in Sox6 protein levels and a decrease of *Myh7* transcription

**Figure 4:**
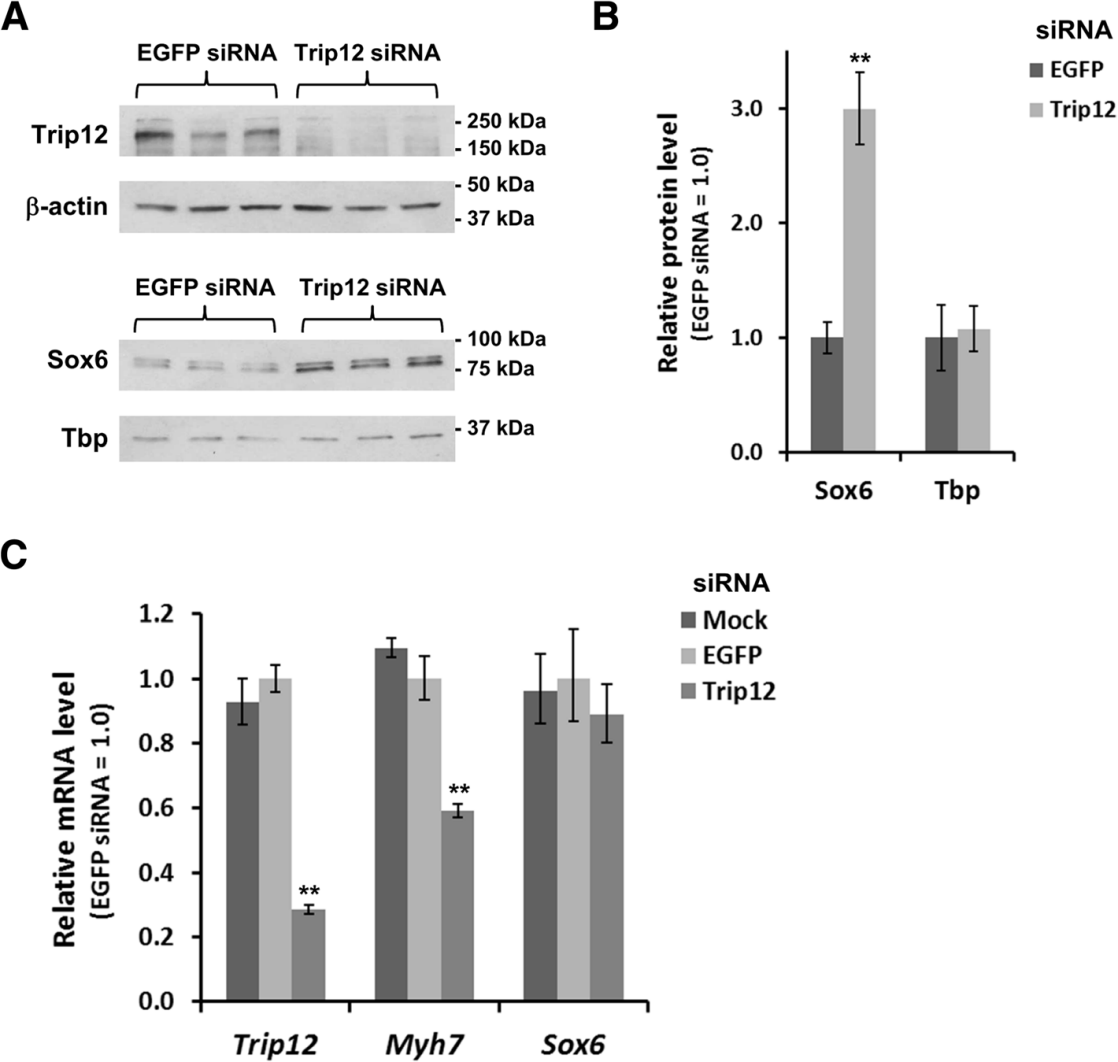
**Trip12 controls Sox6 protein level in C2C12 cells. (A)** siRNA-mediated knockdown of Trip12 resulted in an increase of Sox6 protein levels in C2C12 cells. C2C12 cells were transfected with siRNA for either EGFP (negative control) or Trip12 in triplicate, and lysates were analyzed by Western blotting using a 7.5% gel. **(B)** Densitometric analysis of the Western blot in (**A**) shows approximately 3-fold increase in the Sox6 protein level in Trip12 siRNA-treated cells, while the Tbp protein level was not affected. Data are normalized for those from EGFP siRNA-transfected cells and represented as mean ± SD (*n* = 3). **(C)** Trip12 knockdown lowered mRNA level of *Myh7*, a known Sox6 target. Total RNA was extracted from mock- or siRNA-transfected C2C12 cells, and mRNA levels of *Trip12* and *Myh7* were quantified by reverse transcription-quantitative PCR (RT-qPCR). Data are normalized for those from EGFP siRNA-transfected cells and represented as mean ± SD (*n* = 3). **p < 0.005.

Since polyubiquitination frequently leads to degradation of the target substrate, we next tested the effect of siRNA-induced Trip12 knockdown on Sox6 protein levels in muscle cells. As shown in Figure 4A, in the presence of Trip12 siRNA, intensity of the ~224 kDa band (full-length mouse Trip12) in C2C12 myotubes decreased to almost an undetectable level. Using this Trip12 siRNA, the result of Trip12 inhibition on Sox6 protein levels was determined at a time point (48 h after switching to DM) where Sox6 protein levels are known to be decreasing in differentiating C2C12 myotube cultures (see Additional file 2: Figure S1).

As can be seen in Figure 4B, reduction of Trip12 led to a threefold increase in the amount of Sox6 protein, whereas there was no change in protein levels of the nuclear transcription factor TATA-box binding protein (Tbp) (used as a control). Therefore, Sox6 is a specific substrate to Trip12, by which it is polyubiquitinated and degraded. Whether a Trip12-mediated change in Sox6 protein level had any significant biological effects was initially tested by analysis of changes in mRNA expression of *Myh7*, a direct Sox6 target gene [9,10]. When Trip12 siRNA treatment effectively reduced Trip12 mRNA levels to approximately one third of the control level (Figure 4C), *Myh7* mRNA levels were reduced by 40%, while Sox6 mRNA levels were unchanged (Figure 4C), confirming the suppressive role of Sox6 on the *Myh7* transcription at the post-transcriptional level.

### Sox6 protein is degraded by the 26S proteasome

The final stage of the ubiquitin-proteasome system is degradation of the polyubiquitinated substrate by the 26S proteasome [13]. Therefore, we determined the effect of proteasome inhibition on the cellular Sox6 protein levels.

**Figure 5:**
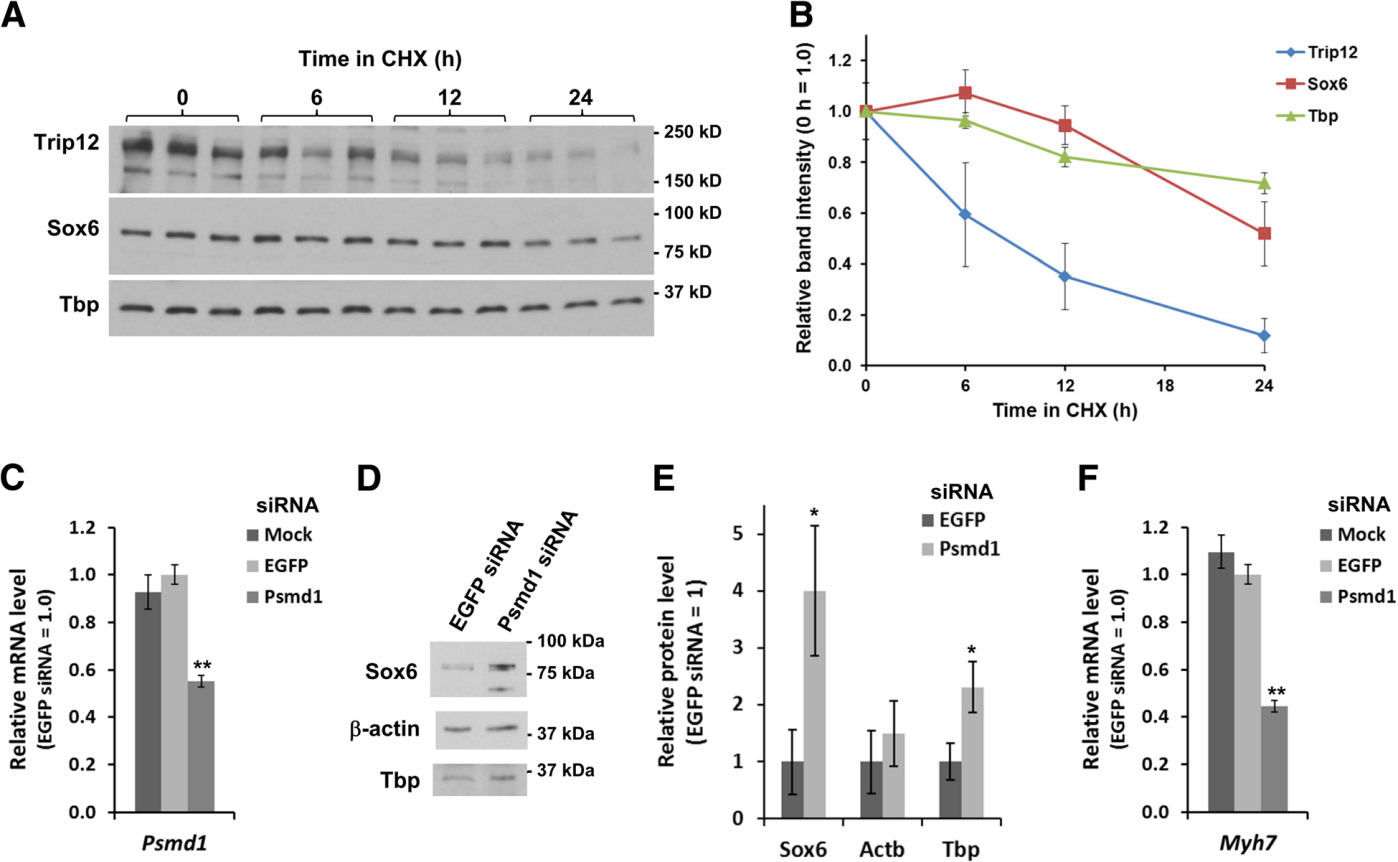
**Sox6 is degraded by proteasome in C2C12 cells.** (**A**) Time course of Trip12, Sox6, and Tbp protein levels in C2C12 cells in the presence of cycloheximde (CHX) analyzed by Western blotting using a 4-15% gradient gel (*n* = 3). (**B**) Densitometric analysis of the Western blot in (**A**). Band intensity of each protein was normalized to that of 0 h in CHX and represented as mean ± SD (*n* = 3). (**C**) Validation of *Psmd1* knockdown by siRNA. Total RNA was extracted from mock- or siRNA-transfected C2C12 cells, and the mRNA level of *Psmd1* (the gene encoding a regulatory subunit of the 26S proteasome) was quantified by RT-qPCR. Data are normalized to EGFP siRNA-transfected cells and represented as mean ± SD (*n* = 3). (**D**) A Western blot using a 7.5% gel showing an increase in the Sox6 protein level in Psmd1 siRNA-transfected C2C12 cells. (**E**) Densitometric analysis of Western blotting results shows a ~4 fold increase in the Sox6 protein level in Psmd1 siRNA-treated C2C12 cells. A smaller increase was observed for Tbp protein. Data are normalized for those from EGFP siRNA-transfected cells and represented as mean ± SD (*n* = 3). (**F**) *Psmd1* knockdown reduced the mRNA level of *Myh7*, a known target of Sox6. Data are normalized to EGFP siRNA-transfected cells and represented as mean ± SD (*n* = 3). **p < 0.005.

A standard approach for examining proteasome-mediated degradation of proteins is to use a combination of a proteasome inhibitor such as MG132 and the protein synthesis inhibitor cycloheximide (CHX) [36]. Prior to this experiment, the stability of the Sox6 protein along with Trip12 and Tbp in C2C12 myotubes was determined. C2C12 myotube cultures (induced for 24 h in DM) were treated with CHX to stop new protein synthesis, following which, the stability of the Sox6, Trip12, and Tbp proteins was examined using Western blot. After 24 h of CHX treatment, the myotubes looked healthy and morphologically normal. As shown in Figure 5A and summarized in Figure 5B, Sox6 and Tbp appeared more stable than Trip12. The protein half-life of Trip12 was estimated as ~9 h, whereas Sox6 protein’s half-life was estimated as ~24 h (Figure 5B). The Tbp protein appeared slightly more stable than Sox6 (Figure 5B). The Trip12 half-life estimated here is in agreement with a similar turnover rate of the human TRIP12 protein recently estimated in HeLa cells using a proteomic approach [37].

Although the Sox6 half-life was found to be relatively long (~24 h), we first tried a conventional proteasome inhibitor (MG132) to examine proteasomal degradation of Sox6. Since extended treatment with proteasome inhibitors is toxic to the cells [38], C2C12 cells were treated with CHX in the absence or presence of MG132 only for 6 h, and the Sox6 protein level was examined by Western blotting. As shown in Additional file 3: Figure S2B, protein levels of myogenin, a short-lived transcription factor [39], were reduced by CHX alone and were recovered by MG132 addition, verifying the effect of MG132 in our experimental condition. To conduct a detailed examination of the response of Sox6 protein to MG132, we used a gel condition optimized for resolving proteins of 75–100 kD (7.5%) and detected two closely sized bands reacting with the Sox6 antibody (Additional file 3: Figure S2B). Although MG132 did not change the overall amount of the Sox6 proteins (total intensity of the two bands; densitometric analysis also showed no statistical difference between these samples: data not shown), there was a significant increase in the upper band along with a significant decrease in the lower band (Additional file 3: Figure S2B), indicating that there is a discrete shift in the Sox6 protein size in the presence of MG132. We found that this size difference was caused by phosphorylation of the Sox6 protein (Additional file 3: Figure S2C). These results suggest a possibility that stability of the Sox6 protein could be regulated by the balance between phosphorylated and non-phosphorylated Sox6 proteins. We are currently investigating this possibility. Because treatment with a conventional proteasome inhibitor did not indicate proteasomal degradation of Sox6 in an unambiguous manner as expected given its long half-life, we next used a siRNA targeting a proteasome subunit to confirm proteasomal degradation of Sox6. We chose to knockdown Psmd1, a subunit of the lid domain of the 26S proteasome, because knockdown of Psmd1 effectively inhibits proteasome activity without significant toxicity [40]. Using *Psmd1* siRNA, we successfully obtained close to 50% reduction in *Psmd1* mRNA levels (Figure 5C). Under this condition, levels of Sox6 proteins significantly increased (Figures 5D and 5E). As shown in Figure 5F, *Psdm1* siRNA treatment of C2C12 myotubes also resulted in significant reduction of *Myh7* mRNA levels. Taken together, these results indicate that Sox6 is degraded by the 26S proteasome and that inhibition of the 26S proteasome activity by siRNA resulted in increased protein levels of Sox6 and increased suppression of its target gene *Myh7* in C2C12 myotubes.

### Knockdown of Trip12 expression results in opposite changes in expression of fast and slow MyHC genes in C2C12 myotubes

Because both Trip12 knockdown and 26S proteasome inhibition in myotube cultures resulted in increased Sox6 protein levels (Figures 4A, 4B, 5D and 5E), and decreased mRNA levels of the Sox6 target *Myh7* (Figures 4C and 5F), we hypothesized that knockdown of Trip12 would result in fiber type-specific gene expression changes opposite to those observed in the Sox6 KO mouse, i.e., an increase in fast fiber-specific gene expression and a decrease in slow fiber-specific gene expression [9,12].

**Figure 6:**
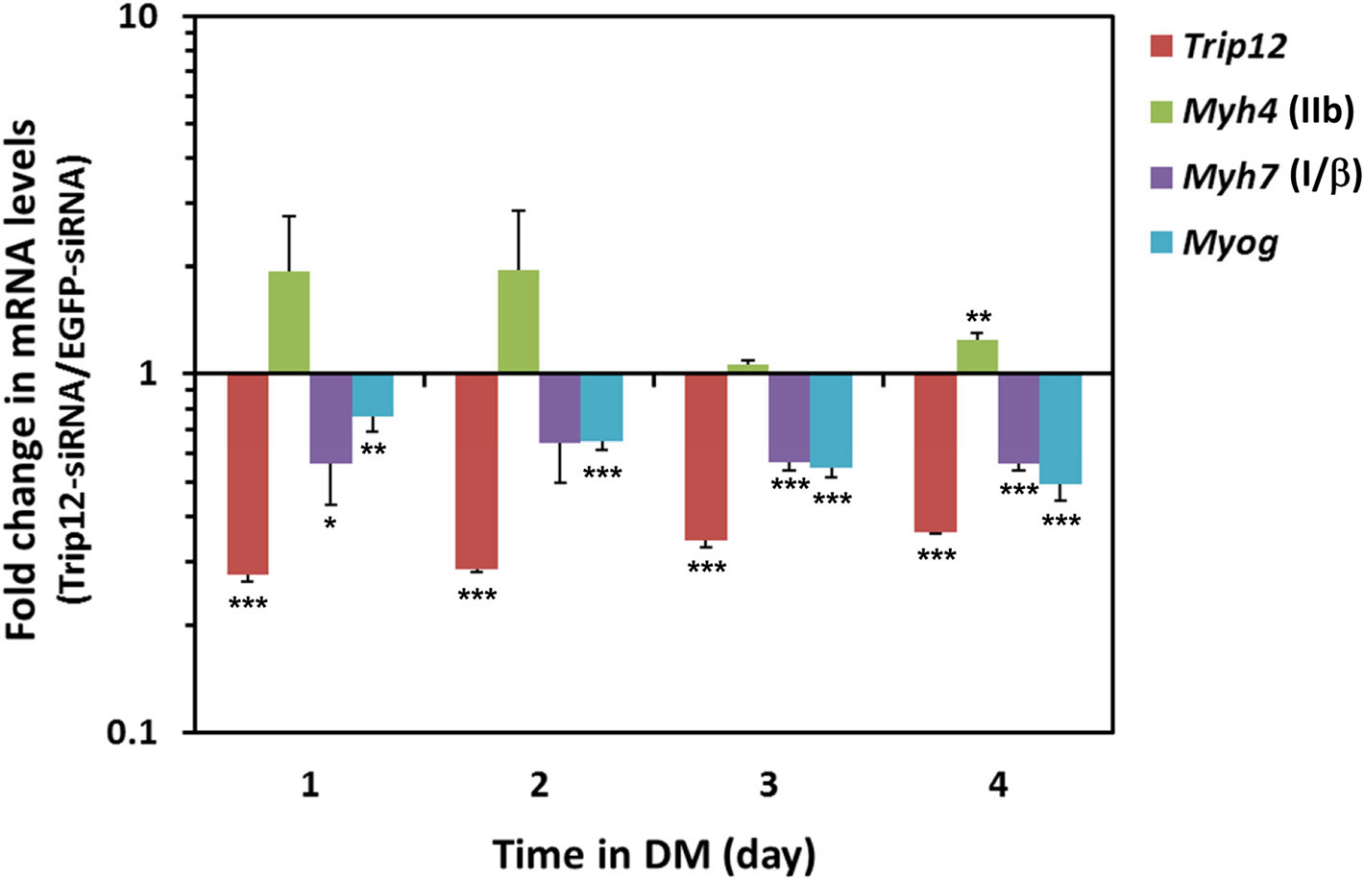
**Trip12 modulates mRNA levels of fiber-type-specific genes in differentiating C2C12 cells.** C2C12 cells were transfected with siRNA for either EGFP (negative control) or Trip12, and medium was switched to DM 24 h after transfection to induce differentiation into myotubes. Total RNA was then extracted every 24 h, and the time course of mRNA levels of *Trip12*, *Myh4* (MyHC-IIb), *Myh7* (MyHC-I/β), and myogenin *(Myog)* were quantified by RT-qPCR using *Huwe1* and *Tbp* as reference genes (see the Methods section for details). Data are normalized for those from EGFP siRNA-transfected cells and represented as mean ± SD (*n*=3). No expression change between EGFP siRNA- and Trip12 siRNA-transfected samples is expressed as 1 on the graph. *p < 0.05, **p < 0.01, ***p < 0.005.

To test this hypothesis, C2C12 myoblasts were transfected with Trip12 siRNA or EGFP siRNA (a negative control) and the mRNA levels of *Myh7* (slow isoform), myogenin (*Myog*: preferentially expressed in slow muscle [41]), and *Myh4* (fast MyHC-IIb isoform) were compared in differentiating C2C12 myotube cultures. Brown et al. [42] showed that in differentiating C2C12 cells, *Myh7* increases after day 1 and then begins to decline after day 4; conversely, *Myh4* begins to increase after day 4 in differentiation medium. In this experiment, we normalized mRNA expression levels in Trip12 siRNA transfected myotubes against the control siRNA transfected myotubes. Changes outside the control expression levels are reflected as greater or lesser than 1. As shown in Figure 6 and Additional file 4: Figure S3, transfection of Trip12 siRNA caused a significant decrease in both *Myh7* and *Myog* mRNA levels as well as a significant increase in *Myh4* (day 4 in DM), a mirror image of their expression patterns in the Sox6 knockout muscle [9,12], suggesting that Trip12 regulation of Sox6 can have a fundamental impact on the fiber type identity.

## Discussion

We have previously reported that a primary mechanism in fiber type-specific gene expression involves the coordinated suppression of hundreds of slow fiber-specific genes by the transcription factor Sox6 [9]. Muscle-specific knockout of Sox6 results in a dramatic increase in slow MyHC-β (*Myh7*) expression coupled with a significant decrease in fast MyHC-IIb (*Myh4*) expression [9,12]. Muscle-specific loss of Sox6 effectively shifts muscle tissue into the slower fiber phenotype leading to the prediction that regulation of Sox6 activity in skeletal muscle could be a focal point of fiber type differentiation. Our present study expands our investigations into the mechanisms of fiber type determination by looking at the regulation of Sox6 by the ubiquitin-proteasome pathway. The identification of the relatively unexplored E3 ligase Trip12 as a Sox6 partner protein led us to investigate the impact of Trip12 activity on cellular Sox6 protein levels. Using siRNA of Trip12, we were able to show that the knockdown of Trip12 expression in C2C12 myotubes resulted in (1) an increase in Sox6 protein levels, (2) the simultaneous downregulation of both the slow fiber-specific *Myh7* and preferentially-slow myogenin, and (3) upregulation of *Myh4* (fast), thus suggesting that Trip12, by controlling Sox6 protein levels, plays a critical role in regulating muscle fiber type-specific gene expression. This observation significantly expands the role of E3 ligases in skeletal muscle beyond their traditionally conceived role in atrophy and, possibly, into the realm of fundamental developmental processes in the muscle.

The role of Sox6 as a transcriptional suppressor of slow fiber-specific isoform genes in skeletal muscle has been reported in mice and zebrafish [9,10,43]. These reports indicate that the function of Sox6 during skeletal muscle development is likely conserved through vertebrate evolution since the loss of Sox6 activity in both species results in the increased mRNA expression of slow fiber-specific genes [9,12,43]. Sox6 is also expressed in the adult heart at a moderate level [17,34]. During heart development, it has been shown that Sox6 is expressed at a high level in proliferating cardiomyocyte progenitor cells, and a reduced Sox6 expression level causes the progenitors to exit from the cell cycle and differentiate [44]. Therefore, control of the cellular levels of the Sox6 protein is potentially a vital mechanism to regulate development of both cardiac and skeletal muscle.

To identify Sox6 interacting proteins, we performed yeast two-hybrid screening and identified TRIP12, an E3 ubiquitin ligase, as a SOX6-interacting protein. In adult mouse tissues, the Trip12 protein is expressed highest in testis and moderately in both skeletal muscle and the heart (Figure 2A). Endogenous Trip12 and Sox6 proteins in C2C12 cells directly interact (Figure 2B), and Trip12 polyubiquitinates Sox6 both *in vitro* and *in vivo* (Figure 3). Ubiquitination is a complex and multifaceted regulatory system involving the modification of a target protein by addition of either one or multiple ubiquitin molecules leading to many different outcomes depending on the number of ubiquitins added or the topology of the ubiquitin chain [14,45]. The addition of one or more ubiquitin molecules to a protein is a multistep process involving at least three ubiquitin enzymes, E1, E2 and E3. In this article, we focused on Trip 12, an E3 ubiquitin ligase, which polyubiquitinates Sox6. The E3 ligases’ role in the ubiquitination chain comes at the end of a multistep process, where it functions to attach one or more ubiquitin molecules to a specific target protein [13,45]. Currently, it is estimated that more than 1,000 distinct E3 ligase genes are encoded in the human genome [13]. The majority of these are classified into the RING family of E3 ligases with a much smaller family of genes belonging to the HECT family, of which Trip12 is a member [28,46]. The HECT domain consists of approximately 350 amino acids encoding the E3 ligase catalytic domain. It is located at the carboxyl terminus and is evolutionarily highly conserved [28,45]. Amino acid sequences lying outside of the HECT domain vary among the family members leading to suggestions that this N-terminus part of the protein primarily determines substrate specificity [28,46]. In the ubiquitination of target proteins, many E3 ubiquitin ligases possess the capability of polyubiquitination, while some are only capable of monoubiquitination when alone, and require an E4 ligase for attachment of additional ubiquitin molecules [35]. Our *in vitro* ubiquitination assay results show that TRIP12 can polyubiquitinate Sox6 in the absence of an E4 (Figure 3A). Interestingly, the length of smear bands detected was different between *in vitro* and *in vivo*: while *in vitro* ubiquitination assay showed smear bands up to ~250 kDa (Figure 3A), a higher molecular weight of smear bands was detected in *in vivo* ubiquitination assay (Figure 3B). These results suggest that there might be additional factor(s) *in vivo* (e.g., an E4 ligase), which assists further elongation of polyubiquitin chains by Trip12. TRIP12, a more recently recognized E3 ubiquitin ligase, was identified based on its sequence homology to the yeast UFD4 E3 ubiquitin ligase [32]. The E3 ligase activity of TRIP12 HECT domain was subsequently demonstrated [18]. The TRIP12 substrates known to date include APP1-BP1 (amyloid beta precursor protein-binding protein 1) [32], the SWI/SNF chromatin remodeling complex subunit BAF57 [31], the tumor suppressor protein ARF [30], and the RING finger E3 ligase RNF168 [47]. Our current report adds the transcription factor Sox6 as a TRIP12 substrate polyubiquitinated in muscle cells. Since the TRIP12 clone identified by yeast two-hybrid screening contained only the HECT domain (Figure 1B) and the TRIP12 HECT domain alone could interact with the SOX6 coiled-coil domain (Figure 1D), we speculate that the HECT domain is the minimal necessary requirement for TRIP12 to recognize SOX6 as a substrate. In fact, the dual role of the TRIP12 HECT domain (E3 ligase catalytic activity and the substrate recognition) has been previously demonstrated [18]. Park and colleagues reported that the HECT domain of TRIP12, but not UBE3A (E6-AP), could recognize and ubiquitinate an E3 ligase substrate on its own [32]. Our current result indicates that this observation holds for the recognition of SOX6 as a substrate by TRIP12. The ability of the HECT domain to function as a ligase catalytic domain as well as a substrate recognition module could be a unique trait for the TRIP12 HECT domain, which will be tested in the future as more information becomes available for the other HECT domain E3 ligases.

The addition of one or more ubiquitin molecules to a target protein lends a tremendous flexibility to the cellular fate of that protein. Depending on the ubiquitin code (for review, see Komander et al. [45]), the activity, location, partners and even the protein’s survival can be altered. We found that Sox6 is polyubiquitinated by TRIP12 and is degraded by the 26S proteasome. In contrast to other critical myogenic regulatory factors such as MyoD or myogenin, the half-life of Sox6 is surprisingly long (~24 h). Half-lives of MyoD, Myf5, and myogenin have been reported as 0.8-1.0 h, <1.0 h, and ~1.0 h, respectively [39,48,49]. The stable nature of Sox6 may reflect its function in muscle development; Sox6 plays a role in specification of myotube phenotype by regulating fiber type differentiation and is expressed at high levels in myotubes [10]. Therefore, the relative stability of Sox6 may be necessary for stably maintaining the fiber phenotype of myotubes. The ubiquitously expressed nuclear protein Tbp is also stable, supporting the correlation of protein stability with maintenance of gene expression.

In muscle, the overall stability of a multigene regulator such as Sox6 lends stability to a phenotype such as muscle fiber type, but this same stability could quickly become a hindrance when external signals demand a fiber type shift. Thus, the polyubiquitination of Sox6 by the Trip12 E3 ligase leading to its degradation would allow for a swift response in the face of changing workloads of the muscle. Therefore, Trip12 likely functions as a pivot point in fiber type transition, altering the balance between slow and fast fiber gene expression. To date, two other mechanisms regulating Sox6 protein activities within the cells have been reported: regulation by microRNA and sumoylation. The Sox6 mRNA has a long (~5 kb) 3’-UTR sequence, which contains multiple microRNA seed sequences [22]. MicroRNAs are expressed in a cell type-specific manner and are known to regulate the protein levels of cell type-specific genes [50]. miR-499 is known to target the Sox6 mRNA and suppresses Sox6 protein expression in skeletal muscle [51-54] and in differentiating cardiomyocytes [44]. In the central nervous system, it has been shown that miR-219 targets Sox6 in oligodendrocytes and induces the terminal differentiation of oligodendrocytes [55,56]. Post-translational modification can also control the function of a protein. In the case of Sox6, it has been shown that SUMO (small ubiquitin-related modifier) reduces the binding affinity of Sox6 to DNA, thus changing its transcriptional activity [57].

In the current report, we have demonstrated that Trip12 ubiquitinates the Sox6 protein, Sox6 is degraded by the 26S proteasome, and in both these instances, the expression of Sox6 target genes are affected. Since skeletal muscle is highly plastic in nature and in transitions between the slow and fast fiber types [58], the speedy removal of relevant transcription factors (e.g., Sox6) via the ubiquitin-proteasome system could allow for a rapid response to external cues.

Taken together, regulation of Sox6 protein activity, both post-transcriptional and post-translational, has critical outcomes on differentiation of various cell types. Although Sox6 mRNA is transcribed in multiple tissues at moderate to high levels [17,34], its role as a transcription factor is quite distinct in different cell types [22]. This is because, in part, cell type-specific Sox6 functions are dictated by its regulatory co-factors [22,24]. To achieve developmental stage-specific functions, however, close monitoring of the Sox6 protein expression level is also critical. In light of this, the ubiquitin-proteasome system mediated degradation of the Sox6 protein likely plays a crucial role in temporal regulation of the Sox6 protein level during development. It is possible that a different set of E3 ligases catalyzes ubiquitination of the Sox6 protein depending on different cellular environments (e.g.. different cell types) or different signals. Therefore, further uncovering of the ubiquitin-proteasome regulation of transcription factors will enrich our understanding of the multi-layered mechanisms of transcriptional regulation during development.

## Conclusions

We have shown here that Trip12, a HECT domain E3 ubiquitin ligase, targets Sox6, a suppressor for slow fiber-specific genes. In addition, we showed that Trip12 is involved in regulation of the fast MyHC isoform gene expression. Based on our current data, we propose that in skeletal muscle, E3 ligases have a significant role in regulating fiber type-specific gene expression, expanding their functional importance in muscle beyond their well-established role in atrophy.

## Abbreviations

CC: Coiled-coil; Co-IP: Co-immunoprecipitation; CHX: Cycloheximide; DM: Differentiation medium; GM: Growth medium; HECT: Homologous to the E6-AP carboxyl terminus; IP: Immunoprecipitation; MyHC: Myosin heavy chain; qPCR: Quantitative PCR; RT-qPCR: Reverse transcription-quantitative PCR; siRNA: Small interfering RNA; WB: Western blot.

## Competing interests

The authors declare no competing interests.

## Authors’ contributions

CIA performed Co-IP, *in vitro* and *in vivo* ubiquitination assays, siRNA experiments, cycloheximide and MG132 experiments, RT-qPCR, Western blotting, and densitometric analysis and contributed to writing the manuscript. EG carried out yeast two-hybrid screening to identify TRIP12 and vector construction. NH conceived, designed, and supervised the study and contributed to writing the manuscript. All authors read and approved the final manuscript.

## Supplementary Material

Additional file 1: Table S1 siRNAs, PrimeTime qPCR Assays, and TaqMan Gene Expression Assay used in the experiments. Click here for file

Additional file 2: Figure S1 Temporal changes in Sox6 expression level in differentiating C2C12 cells. **(A)** RNA was prepared every 24 h from C2C12 cells grown in DM at the indicated time. RT-qPCR for *Sox6* was performed using TaqMan Gene Expression Assays (Applied Biosystems). *Gapdh* expression level was used to normalize data. Fold increase in mRNA levels (0 h in DM = 1) was calculated at each time point. Each data set represents four independent RT-qPCR experiments (mean ± SD). **(B)** Crude protein extract prepared from differentiating C2C12 cells (0, 24, 48, and 72 h in DM) was separated on a 7% SDS-PAGE gel (100 μg per well), and Western blotting was performed using Sox6 antibody. A ~90 kDa Sox6 band was detected in all C2C12 cell extracts. The highest Sox6 protein expression was observed at 24 h in DM and then rapidly dropped close to the 0 h level at 48 h in DM. β-actin was used as a loading control. Click here for file

Additional file 3: Figure S2 MG132 treatment shifted the intensity of the two Sox6 bands in differentiating C2C12 cells. **(A)** A procedure for MG132 experiments. C2C12 cells were seeded in six-well plates at a density of 3 × 10^5^ cells/well and incubated in GM for 24 h. Cells were rinsed with PBS once and incubated in DM. After 48 h, 100 μg/ml CHX and 1 μM MG132 or the same volume of DMSO (with which MG132 stock solution was prepared) were added to DM, and cells were incubated for another 6 h before Western blotting. **(B)** Western blots of MG132-treated cells. Three independent samples were prepared for each treatment. Tbp was used as a loading control. **(C)** Phosphatase treatment of C2C12 nuclear protein; 5 μg of nuclear protein prepared from differentiating C2C12 cells without phosphatase inhibitor cocktail was treated with 10 units of calf intestinal alkaline phosphatase (CIP) in the absence or presence of phosphatase inhibitor cocktail (PIC) for 10 min at 37°C and analyzed by Western blotting using a 7.5% gel. Positions of two Sox6 bands, which are always seen on 7.5% gels but not on 4-15% gradient gels, are indicated. CIP treatment shifted the position of Sox6 band from upper to lower size, indicating that the upper band consists of a phosphorylated form of Sox6. Click here for file

Additional file 4: Figure S3 Relative mRNA levels of fiber-type-specific genes shown in Figure 6. Relative mRNA levels against reference genes (*Huwe1* and *Tbp*) were calculated using the formula 2^-ΔCt^ and represented as mean ± SD (*n*=3). Click here for file
