# Supplementary figures and images for: Trip12, a HECT domain E3 ubiquitin ligase, targets Sox6 for proteasomal degradation and affects fiber type-specific gene expression in muscle cells
Source: Skelet Muscle. 2013 May 10;3:11. doi: 10.1186/2044-5040-3-11 (PMC3666947; doi:10.1186/2044-5040-3-11)

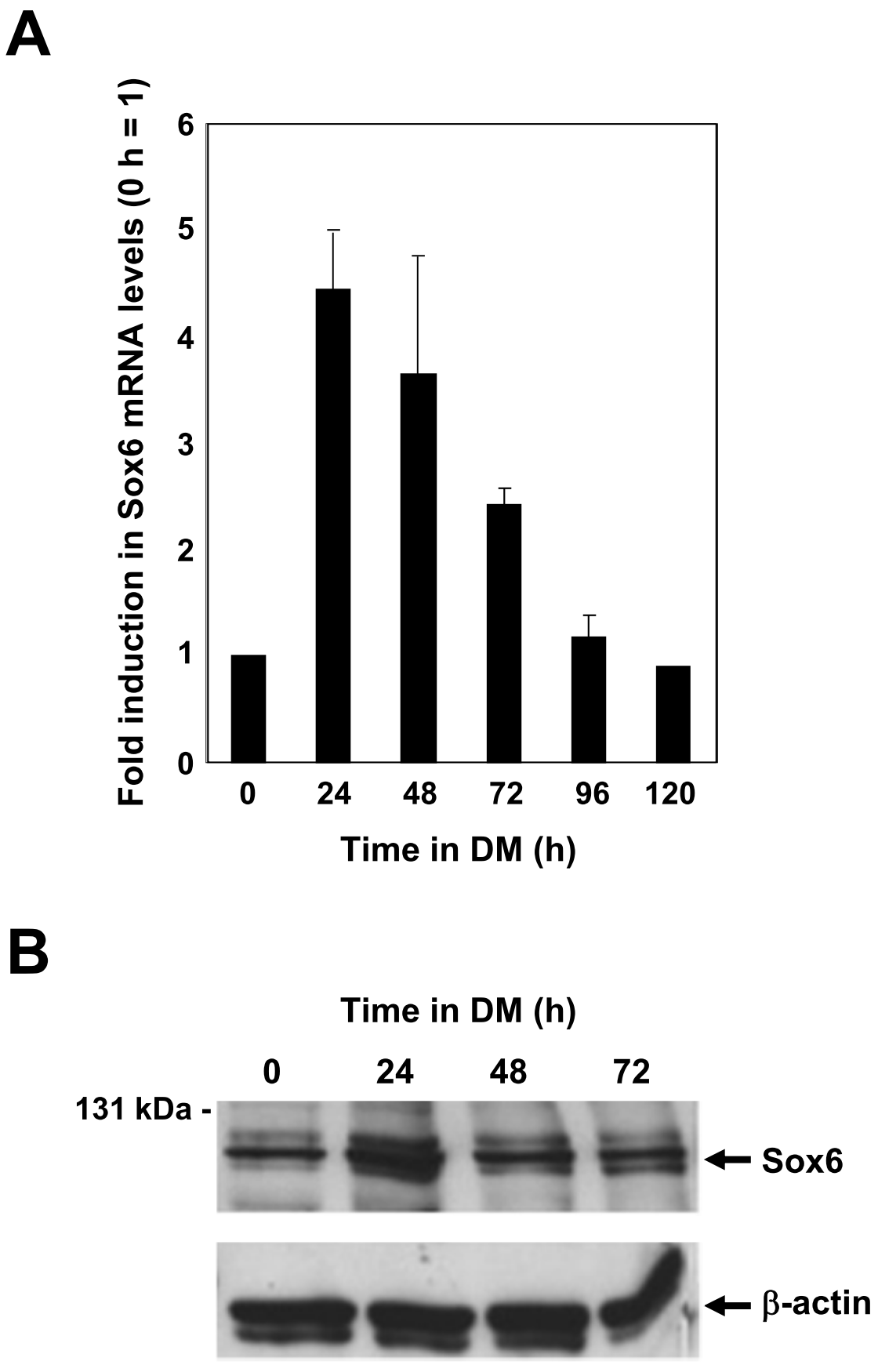

Supplement: Additional file 2: Figure S1 — Temporal changes in Sox6 expression level in differentiating C2C12 cells. (A) RNA was prepared every 24 h from C2C12 cells grown in DM at the indicated time. RT-qPCR for Sox6 was performed using TaqMan Gene Expression Assays (Applied Biosystems). Gapdh expression level was used to normalize data. Fold increase in mRNA levels (0 h in DM = 1) was calculated at each time point. Each data set represents four independent RT-qPCR experiments (mean ± SD). (B) Crude protein extract prepared from differentiating C2C12 cells (0, 24, 48, and 72 h in DM) was separated on a 7% SDS-PAGE gel (100 μg per well), and Western blotting was performed using Sox6 antibody. A ~90 kDa Sox6 band was detected in all C2C12 cell extracts. The highest Sox6 protein expression was observed at 24 h in DM and then rapidly dropped close to the 0 h level at 48 h in DM. β-actin was used as a loading control. [file 2044-5040-3-11-S2.tiff]

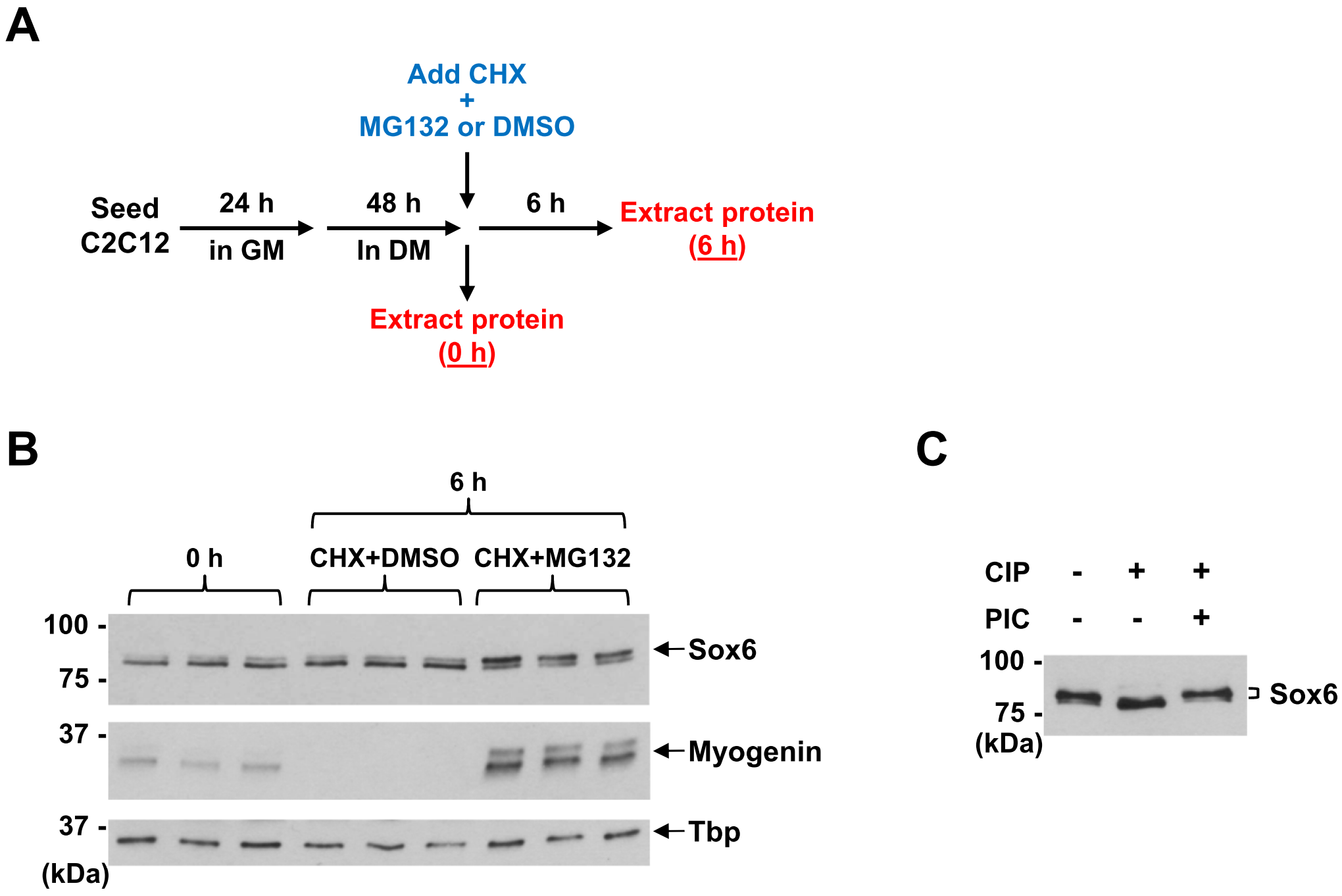

Supplement: Additional file 3: Figure S2 — MG132 treatment shifted the intensity of the two Sox6 bands in differentiating C2C12 cells. (A) A procedure for MG132 experiments. C2C12 cells were seeded in six-well plates at a density of 3 × 10^5^ cells/well and incubated in GM for 24 h. Cells were rinsed with PBS once and incubated in DM. After 48 h, 100 μg/ml CHX and 1 μM MG132 or the same volume of DMSO (with which MG132 stock solution was prepared) were added to DM, and cells were incubated for another 6 h before Western blotting. (B) Western blots of MG132-treated cells. Three independent samples were prepared for each treatment. Tbp was used as a loading control. (C) Phosphatase treatment of C2C12 nuclear protein; 5 μg of nuclear protein prepared from differentiating C2C12 cells without phosphatase inhibitor cocktail was treated with 10 units of calf intestinal alkaline phosphatase (CIP) in the absence or presence of phosphatase inhibitor cocktail (PIC) for 10 min at 37°C and analyzed by Western blotting using a 7.5% gel. Positions of two Sox6 bands, which are always seen on 7.5% gels but not on 4-15% gradient gels, are indicated. CIP treatment shifted the position of Sox6 band from upper to lower size, indicating that the upper band consists of a phosphorylated form of Sox6. [file 2044-5040-3-11-S3.tiff]

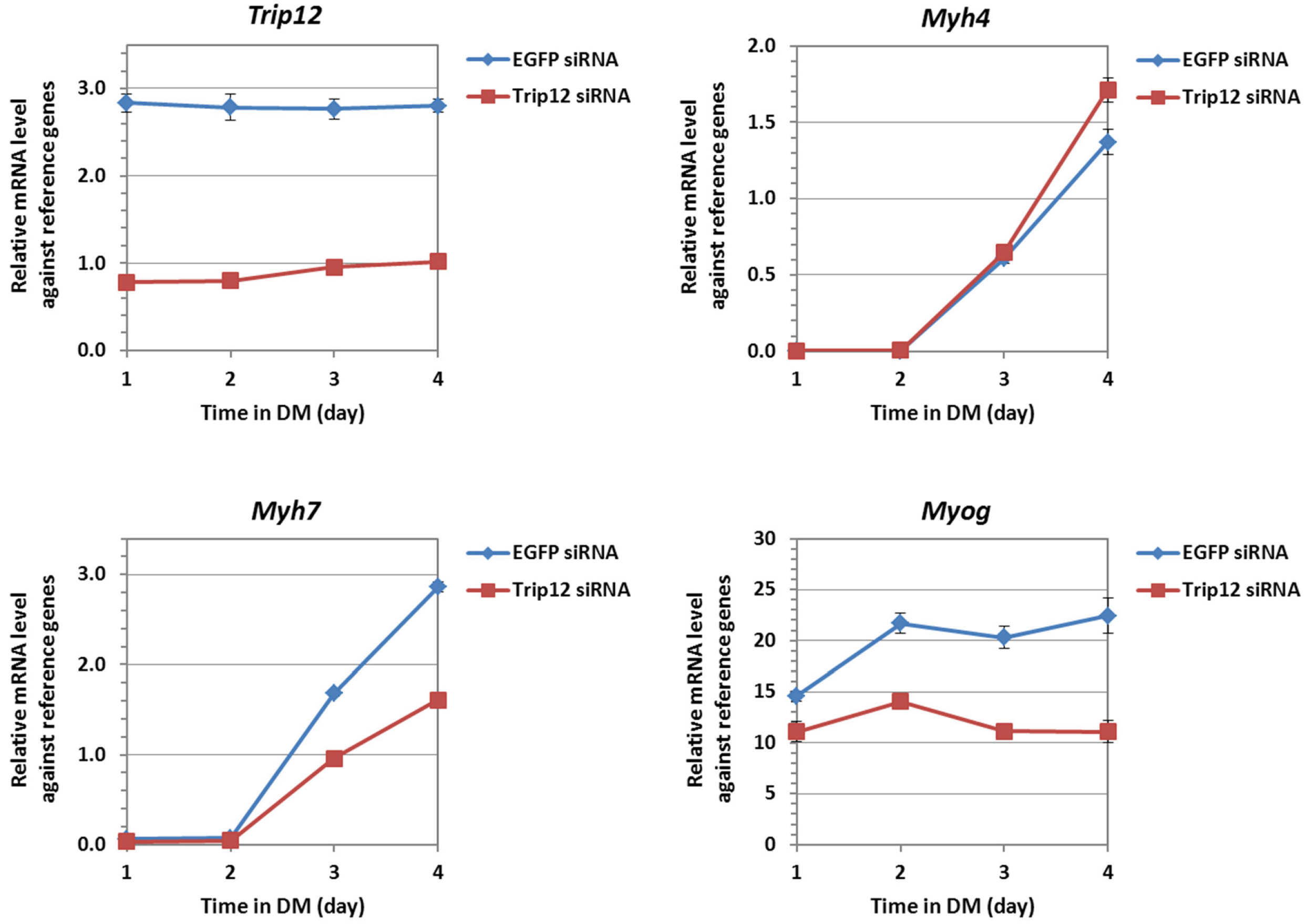

Supplement: Additional file 4: Figure S3 — Relative mRNA levels of fiber-type-specific genes shown in Figure 6. Relative mRNA levels against reference genes (Huwe1 and Tbp) were calculated using the formula 2-ΔCt and represented as mean ± SD (n=3). [file 2044-5040-3-11-S4.tiff]
